# The role of the tissue microenvironment in the regulation of cancer cell motility and invasion

**DOI:** 10.1186/1478-811X-8-22

**Published:** 2010-09-07

**Authors:** Jan Brábek, Claudia T Mierke, Daniel Rösel, Pavel Veselý, Ben Fabry

**Affiliations:** 1 Department of Cell Biology, Faculty of Science, Charles University, Prague, Czech Republic; 2 Biophysics Group, Department of Physics, University of Erlangen-Nuremberg, Germany; 3 Institute of Molecular Genetics, AS CR, Prague, Czech Republic

## Abstract

During malignant neoplastic progression the cells undergo genetic and epigenetic cancer-specific alterations that finally lead to a loss of tissue homeostasis and restructuring of the microenvironment. The invasion of cancer cells through connective tissue is a crucial prerequisite for metastasis formation. Although cell invasion is foremost a mechanical process, cancer research has focused largely on gene regulation and signaling that underlie uncontrolled cell growth. More recently, the genes and signals involved in the invasion and transendothelial migration of cancer cells, such as the role of adhesion molecules and matrix degrading enzymes, have become the focus of research. In this review we discuss how the structural and biomechanical properties of extracellular matrix and surrounding cells such as endothelial cells influence cancer cell motility and invasion. We conclude that the microenvironment is a critical determinant of the migration strategy and the efficiency of cancer cell invasion.

## Introduction

The malignancy of cancer is rooted in the ability of tumor cells to spread to distant locations in the body and to form metastases. The formation of metastases is a complex process involving multiple steps: first, tumor cells must break away from the primary tumor and invade through the surrounding tissue and its extracellular matrix (ECM). Matrix invasion is one of the earliest steps in the metastatic process and a key determinant of the metastatic potential of tumor cells. Next, the tumor cells enter the bloodstream or the lymph vessel system which enables them to quickly and efficiently spread to distant sites; therefore, the metastasizing tumor cells must be capable of intravasation, survival in the bloodstream or lymphatic system, and extravasation (reviewed in [1]). Regardless of whether extravasation takes place, however, the migration through connective tissue (subsequently called invasion) is a prerequisite for metastasis formation.

Although cell invasion is foremost a mechanical process, cancer research has focused largely on gene regulation and signaling that lead to uncontrolled cell growth. More recently, the genes and signals involved in the invasion and transendothelial migration of cancer cells, such as the role of adhesion molecules and matrix degrading enzymes, have become the focus of research [2-4]. However, the mechanical processes themselves that control cancer cell invasion, such as cell adhesion, changes of cell shape, cell movements and motility, and the generation of forces, are currently not well understood [5-8]. We argue that the invasion process can only be understood in the context of the cancer cells' interactions with its environment. In this review we discuss how the structural and biomechanical properties of the extracellular matrix and surrounding cells such as endothelial cells influence cancer cell motility and invasion strategies.

## Biophysical properties of the ECM and their influence on cancer cell motility

The connective tissue forms a mechanically stable support for epithelial cells, permits the diffusion of oxygen and nutrients between the microvasculature and adjacent tissues, and enables the trafficking of freely moving cells. The connective tissue is composed of a biopolymer fiber network of proteins, proteoglycans and glycosaminoglycans that differ in composition and structure throughout various parts of the body. The size of the biopolymer fibers and the density of the fiber network determine the mechanical, or rheological, properties as well as the morphological properties of the ECM such as porousness and mesh size.

### Matrix Morphology

The fiber network morphology has a direct impact on how much resistance a moving cell encounters. If the cross-section of the migrating, elongated cell matches or is slightly below the mesh size, then the cell encounters no resistance, or steric hindrance. If the mesh size is too large, the migration speed decreases [9] due to a loss of cell-fiber attachment sites that are needed to either push or pull the cell body forward. But as we will see below, there are also other secondary, less direct effects of fiber density on cell migration behavior.

Large mesh sizes make it possible that cells with a round cell shape can migrate through the network in a so-called amoeboid fashion. If only very few fibers remain, however, the cell is limited to an effective "1D" migration mode [10]. Conversely, if the fiber density increases such that the mesh size becomes too small, the migration speed decreases and the cells may get trapped [9]. Large-scale cell deformability is mostly governed by the rigidity of the nucleus which is regulated by nuclear lamins A/C [11,12]. The cell has several options to move through a pore that is smaller than its cross section. The cell can either force the network fibers apart, it can remodel its shape until it can pass through the pore, or it can degrade the fiber matrix with the help of proteolytic enzymes [13,14].

### Matrix Rheology

The force that is needed to move the network fibers apart and "out of the way" is determined by the mechanical, also called rheological, properties of the ECM. These include the frequency-, strain- and strain-rate-dependent visco-elastic shear modulus, the strain-dependent plasticity, compressibility, and Poisson-ratio. These mechanical parameters are related to the structural and molecular properties of the tissue, in particular collagen content, fiber thickness, and the extent of intrafibrillar cross-links [15].

### Mechanosensing

On the one hand, fiber pore size and mechanical properties determine the passive steric hindrance of the ECM. On the other hand, ECM mechanical properties are sensed by the cell and can lead to an active adaptation so that cells may increase their protrusive forces to compensate for increased steric hindrance of the matrix. This mechano-sensing is facilitated by integrin-mediated adhesions and downstream mechanosensor protein signaling (i.e., via vinculin, talin, FAK, p130CAS and filamin A; [16-20]. Increased stiffness of the surrounding ECM evokes a reinforcement of focal adhesions and increased RhoA-mediated actomyosin contraction, ultimately leading to cell protrusions, high-traction forces, and elongated cell shapes [21]. Conversely, a soft matrix does not lead to focal adhesion reinforcement and cytoskeletal contractility; rather, it encourages cell rounding [22]. In this way, tissue rigidity can stimulate directed cell migration as potently as the presence of a chemotactic gradient. In particular, cells tend to move toward regions of greater stiffness, a process known as durotaxis [23].

The mechanical ECM properties can be changed and remodeld by the activity of tumor cells. Such ECM remodeling leads to the characteristic stiffening of the tumor tissue. The importance of ECM remodeling for cancer progression becomes increasingly more appreciated. Recently, Leventhal and co-authors reported that breast tumorigenesis is accompanied by collagen crosslinking, ECM stiffening, and increased focal adhesion formation [24]. Induction of collagen crosslinking stiffened the ECM, promoted focal adhesions, enhanced PI3 kinase activity, and induced the invasion of an oncogene-initiated epithelium. In contrast, the inhibition of integrin signaling repressed the invasion of a premalignant epithelium. Consistently, reduced matrix stiffness by a reduction of lysyl oxidase-mediated collagen crosslinking impeded malignancy and lowered tumor incidence [24].

### Contact guidance

Connective tissues show different fiber arrangements that can range from loose or random to highly aligned structures [25,26]. Cancer cells display an aligning behavior, called contact guidance, [27] and orient themselves along these structural ECM elements [28]. Contact guidance is mediated by mechanosensory integrins that, together with Rho/ROCK-mediated cytoskeletal orientation and directional contraction, enable the directional persistence in cell invasion [28]. Conversely, matrix fiber alignment and reorganization is also cell contractility-dependent and mediated by Rho/Rho kinase pathway activity [28]. But even when Rho or Rho kinase pathways are inhibited, 3 D cell migration is still enhanced by fiber alignment.

In summary, the structural and mechanical properties of the ECM have a substantial impact on cell behavior. They modulate cell adhesions, cytoskeletal reorganization and cell shape, and through contact guidance mechanisms lead to directed cell migration that is essential for tumor spreading, transendothelial migration and metastasis formation.

## Mechanisms of cancer cell migration through connective tissue

**Figure 1:**
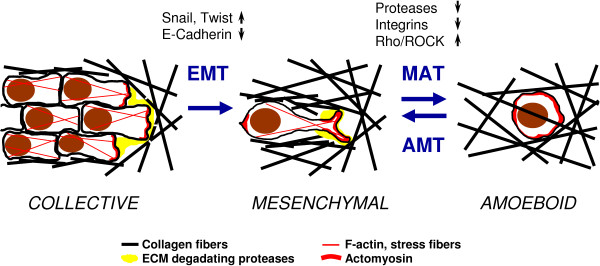
**Transitions among cell invasion modes**. The conversion from epithelial cells to motile mesenchymally migrating cells (EMT) is induced by repression of transcriptional regulators such as Snail or Twist which leads to a downregulation of E-cadherin and consequently to a loss of intercellular junctions. Invasion of individual mesenchymal cells is dependent on proteolytical degradation of the surrounding ECM. The degradation processes localize at the anterior edge of the cell and eventually generate a path for invasion. When integrin activation or extracellular protease activity is decreased in mesenchymal cells, or when Rho/ROCK signaling is upregulated, a transition towards amoeboid movements (MAT) occurs. Conversely, inhibition of Rho/ROCK signaling may result in amoeboid mesenchymal transition (AMT). The mesenchymal cells exhibit elongated morphology in a 3 D environment, with actomyosin contractile units located at the front and rear of the cells, while amoeboid cells typically exhibit a round shape in 3 D matrices, with a more cortical distribution of actomyosin fibers.

In the following, we take a closer look at the process of cancer cell invasion and the different mechanisms and strategies that cancer cells employ to move through connective tissue. Tumor cells can migrate either collectively, retaining their intracellular junctions, or individually. The conversion from epithelial cells to motile individually migrating cells is an intensively studied process known as epithelial-mesenchymal transition (EMT). EMT is induced by repression of transcriptional regulators such as Snail or Twist which leads to downregulation of E-cadherin and consequently to loss of intercellular junctions (reviewed in [1]). Individual cell migration strategies are broadly classified as either mesenchymal or amoeboid (for a review see [29]). Yet the amoeboid and mesenchymal invasion modes are not mutually exclusive, and the suppression or enhancement of specific molecular pathways can induce a mesenchymal-amoeboid transition or amoeboid-mesenchymal transition (Figure 1). While an epithelial-mesenchymal transition is accompanied by extensive alterations in gene transcription and therefore is a relatively slow process, cells can switch rapidly, even within minutes, between amoeboid and mesenchymal invasion strategies, depending on the local ECM environment. However, it should be noted that the bidirectional mesenchymal-amoeboid transition was shown only in vitro, and its relevance in vivo has yet to be confirmed.

### Mesenchymal invasion

**Figure 2:**
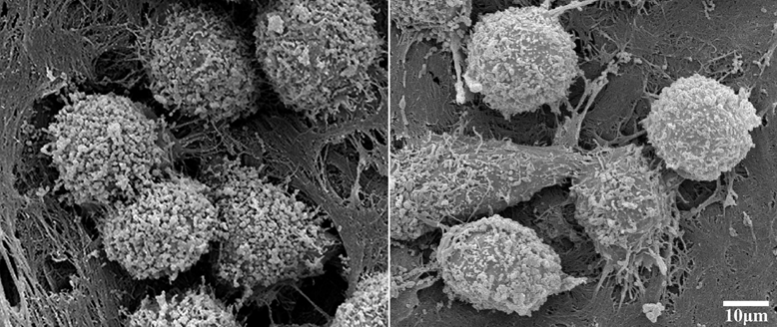
**Scanning electron microscopy image of mesenchymally invading cells**. K4 sarcoma cells were seeded on the acellular dermis [74] in the absence (left) or presence (right) of a broad-spectrum MMP-inhibitor (GM6001). In the absence of the inhibitor, K4 cells extensively degrade the matrix, whereas in the presence of the inhibitor they are not able to invade, and the matrix remains mostly intact.

The mesenchymal type of cell migration resembles fibroblast-like motility and is shared, apart from fibroblasts, by keratinocytes, endothelial cells, macrophages and many tumor cells types. Cells with a mesenchymal type of motility exhibit an elongated spindle-like shape with one or more leading pseudopods. Movement of the mesenchymally migrating cells is initiated by the formation of actin-rich filopodia and lamellipodia at the leading edge. This process is controlled by the small Rho-GTPases Rac and Cdc42 [30,31]. What then follows is a cycle of adhesion to the ECM, stress fiber formation, contraction, and detachment at the rear end of the cell [32]. Moreover, integrin clustering at the leading edge and the associated adhesion structures recruit ECM-degrading enzymes such as MT1-MMP (membrane-type 1 matrix metalloproteinase), cathepsins, and the complex of urokinase-type plasminogen activator (uPA) and its receptor (uPAR), to generate a path for cell migration through an otherwise too dense ECM (Figure 2) [33-35].

### Amoeboid invasion

The term amoeboid migration is inferred from the motility of amoeba, which is characterized by cycles of expansion and contraction of the cell body and bleb-like protrusions mediated by cortically localized actin-myosin interactions [36]. Amoeboid-like movement in higher eukaryotes has been observed and described in leukocytes [37,38] and many types of tumor cells [4,39-42]. Tumor cells that exhibit an amoeboid mode of migration usually show a rounded shape in 3 D substrates.

### Contractile and adhesion forces

The enhanced contractility of cells that use amoeboid-like invasion strategies is facilitated by the activation of the Rho/ROCK pathway and increased phosphorylation of MLC [39,40]. Cortical acto-myosin contraction enables the cells to squeeze through gaps and holes in the ECM structure and to adapt their body shapes to the pre-existing spaces. Thus, unlike path-creating mesenchymally migrating cells, amoeboid cells can be described as path-finding [4,4,37,38]. Amoeboid-like motility requires little or no receptor-facilitated adhesion to the ECM. Moreover, it has been suggested that amoeboid cells could exert sufficient protrusive "pushing" forces to deform the surrounding ECM [28,40,41].

### Matrix remodeling

For a long time, the proteolytic remodeling of ECM by matrix metalloproteinases (MMPs), serine proteases and cathepsins was considered to be a critical determinant of tumor cell invasiveness. Recent data, however, demonstrate that amoeboid-like invasion is proteases-independent [4,40]. In fact, several lines of evidence suggests that cells can undergo a mesenchymal-to amoeboid transition after blocking of pericellular proteolysis [4,39] or blocking of integrins [43]. For instance, mesenchymally migrating HT-1080 fibrosarcoma cells are still able to invade a 3 D collagen matrix after treatment with an inhibitor cocktail that blocked ECM proteolysis. After treatment, these cells show the typical features of amoeboid invasiveness such as rounded morphology and the use of cortical actomyosin contraction during migration [4,40,44]. The induced amoeboid migration of HT-1080 cells after the inhibition of proteolysis is associated with a decreased cell surface expression of α2β1 integrins and a decrease in phosphorylation of focal adhesion kinase (FAK) [44], suggesting a lower requirement for the formation and signaling from focal adhesions. Low affinity adhesions to the substrate and independence on proteolytic degradation of the surrounding ECM enables the amoeboid cells to move in a 3 D environment at relatively high velocities, ranging from 2 μm/min as observed in A375m2 melanoma cells [39] to 25 μm/min, which is also the maximum migration velocity of lymphocytes observed in collagen gels [45]. The failure of MMP inhibitors in recent clinical trials to prevent cancer progression points to the possibility that protease independent mechanisms of invasion may be clinically relevant (reviewed in [46]). Alternatively, non-proteolytic functions of MMPs, the involvment of other proteases than MMPs, or adverse reactions to the inhibitors could also explain this observation.

### Influence of the ECM

Besides blocking of proteolysis and integrins, also the architecture of the ECM, in particular the spatial organization of the collagen fibers, can determine the mode of invasion [28]. To mimic the invasion of tumor cells from the primary tumor into the surrounding matrix, carcinoma cells can be seeded at high cell densities into 3 D collagen gels, and small pieces of this gel-cell mixture are then transplanted into fresh, isotropic, cell-free collagen gels [28]. Under such conditions, the invasion of MDA-MB-231 breast carcinoma cells into the surrounding gel has been reported to be amoeboid-like, protease-independent and driven by Rho/ROCK-mediated contractility. When the gel-cell explants were transplanted into anisotropic collagen gels with aligned fibers and presumably higher stiffness, however, the carcinoma cells migrated in a mesenchymal fashion [28].

ECM architecture-dependent invasion modes have also been observed in human macrophages that either use the amoeboid migration mode in fibrillar collagen-I, or the mesenchymal migration mode in Matrigel and gelled collagen [47]. When migrating mesenchymally in 3 D, the macrophages form proteolytic structures at the tips of cell protrusions that resemble podosome-type adhesion structures. Moreover, when infiltrating matrices of similar composition but with variable stiffness, macrophages adapt their migration mode primarily to the matrix architecture [47].

The relative importance of protease-dependent and -independent invasion modes of cancer cell invasion through interstitial barriers remains a subject of considerable debate. Recently, it has been suggested that the amoeboid invasiveness of tumor cells can only occur under specific conditions that rarely occur *in vivo*, and may not be as effective as mesenchymal cell migration for overcoming the steric hindrance of dense and relatively stiff connective tissue [4,26,39,40]. For instance, the invasiveness of amoeboid HT-1080 and MDA-MB-231 cells after blocking of proteolytic activity was much lower in stiff matrices derived from intact full-length collagen gels as compared to softer pepsin-extracted collagens. The authors conclude that MT1-MMP-independent invasion can only proceeds when the structural pores formed in collagen gel networks are no longer stabilized by the covalent transglutaminase cross-links that normally determine fibril architecture and structural rigidity in full-length collagen. It would be interesting, however, to repeat these experiments with tumor cells that use an amoeboid mode as their primary invasion strategy.

## Endothelial transmigration

How quickly cancer cells are able to migrate through connective tissue in vivo is still debated. In vitro, migration speeds of up to 25 microns per minute through collagen networks have been reported [45]. It is conceivable that cancer cells may actually have years of time during which they can travel for instance along neuronal pathways to distant sites. Such metastatic cancer cells may lie dormant at those sites for a long time until they spring to action and proliferate quickly. The more common view, however, is that cancer cell migration through connective tissue is too slow and undirected to account for the quick spreading and metastasis formation seen in many tumors. Instead, the cancer cells can spread much more quickly and efficiently via lymph or blood vessels to distant sites. Thus, the cancer cells only need to migrate through connective tissue until they reach the nearest blood or lymph vessel [48-50] and then to transmigrate through the endothelial lining and the basement membrane [51-56].

### Role of the endothelium

The endothelial lining and the basement membrane form a passive physical barrier such that the process of intravasation is a potentially time-consuming and rate-limiting step in metastasis formation [50,51,57-59]. But the endothelium can also take an active part in this process and can either support or suppress cancer cell adhesion and possibly their transmigration [53-55,60,61]. How exactly the endothelium functions in this process, however, is still elusive and under investigation. In particular the mechanisms by which cancer cells can transmigrate through the endothelial lining are not well understood.

### Cell-cell signaling

What seems certain, however, is the existence of a crosstalk between cancer cells and endothelial cells. The presence of cancer cells can induce the upregulation of adhesion molecule expression by the endothelium [62], the reorganization of the endothelial acto-myosin cytoskeleton [63], and Src-mediated disruption of endothelial VE-cadherin-*β*-catenin cell-cell adhesions [52]. These processes may either enable paracellular transmigration through the formation of "holes" within the endothelial monolayer [64] and through the induction of endothelial cell apoptosis [65], or they enable transcellular transmigration through regional modulation of cortical acto-myosin generated tension [66].

### Signals from cancer cells

The transmigration process of cancer cells seems to a great extent resemble that of leukocytes. For example, the normal function of the endothelial lining as a barrier against both leukocyte trafficking and cancer cell transmigration [67] is reduced in the presence of inflammatory cytokines such as tumor necrosis factor-α and interleukin-1β [53,62,68,69]. These and other cytokines promote transmigration and invasion by several mechanisms. First, the adhesion molecule E-selectin is upregulated in endothelial cells [62] upon exposure to cytokines. The upregulation of E-selectin subsequently leads to adhesion of leukocytes and cancer cells through E-selectin ligands. Moreover, the adhesion of these cells induces an upregulation of the stress-activated protein kinase-2 (SAPK2/p38) in endothelial cells [62] and induces actin polymerization and stress fiber reorganization [63]. Second, cytokine exposure directly causes cytoskeletal rearrangements in endothelial cells as well as cancer cells and leukocytes, which may prime them for efficient migration. Third, cytokine gradients lead to a more efficient directional migration and invasion in leukocytes [70,71] and cancer cells [72].

### Signals from endothelial cells

It has been recently shown that the endothelial cells themselves are a significant source of chemokines such Gro-β and IL-8 [61]. These chemokines lead to enhanced contractile force generation, cytoskeletal remodeling, and thereby enhanced transmigration and invasion efficiency in cancer cells with high expression levels of the Gro-β and IL-8 receptor CXCR2 [61]. Even more surprisingly, the amount of chemokine secretion by the endothelial cells was greatly modulated by the presence of some but not all cancer cells. This cross-talk between cancer cells and endothelial cells may be in part responsible for the "homing" of certain cancer cell types to specific organs [61].

The reverse process of extravasation, in contrast, need not be a rate-limiting step in metastasis formation since at least some types of cancer cells can adhere and grow within vessels and do not need to extravasate to induce angiogenesis and to form secondary tumors [73]. Even so, the endothelial cells may still impact tumor growth by modulating cancer cell adhesion and by secreting chemokines and growth factors.

## Conclusions

The structural and mechanical properties of extracellular matrix and the presence of signaling molecules from embedded cells have a profound influence on cancer cell motility, tissue invasion, transendothelial migration and metastasis formation. Cancer cells react to their environment through the modulation of cell adhesions, contact guidance, cytoskeletal reorganization, cell shape changes, secretion of proteolytic enzymes and chemokines, and force generation. From insights in this process we expect the development of novel cancer therapies that target the process of metastasis formation by interfering with the ability of cancer cells to transmigrate into blood and lymph vessels and to invade the connective tissue. Cell-matrix-interactions in a 3-dimensional environment, however, are currently not well understood. This is attributable to the difficulty in generating 3-D matrices with controlled morphology, rheology, and matrix composition, and a lack of established methods to visualize and evaluate cell functions over prolonged periods. Similarly, 3-D in-vitro systems to study cancer cell interactions with other cells from the vasculature or immune system, or even to to study 3-D cell behavior in a well-controlled gradient of growth factors or chemokines, are largely missing. These technical and methodological difficulties need to be urgently solved. Only then will we be able to gain a thorough understanding of the interactions between cancer cells and their physical and biochemical environment that is crucial for the development of novel cancer therapies.

## List of abbreviations used

ECM: extracellular matrix; EMT: epithelial-mesenchymal transition; MAT: mesenchymal-amoeboid transition; AMT: amoeboid-mesenchymal transition

## Competing interests

The authors declare that they have no competing interests.

## Authors' contributions

JB, CM, DR and PV all significantly contributed to the writing of the manuscript. BF revised it critically and all authors have given final approval of the version to be published.
